# 

**Published:** 1907-03

**Authors:** 


### BOOKS RECEIVED.

The Practitioner's Medical Dictionary. An Illustrated Dictionary of Medicine and Allied Subjects, Including all the Words and Phrases Generally used in Medicine, with Their Proper Pronunciation, Derivation and Definition. By George M. Gould, A. M., M. D., author of "An Illustrated Dictionary of Medicine, Biology, and Allied Sciences," "The Student's Medical Dictionary," "30,000 Medical Words Pronounced and Defined," etc.; Editor of "American Medicine." With 388 Illustrations. Octavo; xvi-1043 pages. Philadelphia: P. Blakiston's Son & Co. (Flexible leather, gilt edges, rounded corners, $5.00; with thumb index, $6.00, net).

---

A Textbook of Pharmacology. Including Therapeutics, Materia Medica, Pharmacy, Prescription-Writing, Toxicology, etc. By Torald Sollmann, M.D., Assistant Professor of Pharmacology and Materia Medica, Western Reserve University, Cleveland, Ohio. New (2d) edition. Octavo of 1070 pages, fully illustrated. Philadelphia and London: W. B. Saunders Company. 1906. (Cloth, $4.00; halfnorocco $5.00, net prices).

---

Atlas and Textbook of Human Anatomy. Volume II. By Professor J. Sobotta, of Wurzburg. Edited, with additions, by J. Playfair McMurrich, A.M., Ph.D., Professor of Anatomy at the University of Michigan, Ann Arbor. Quarto volume of 194 pages, containing 214 illustrations, mostly all in colors. Philadelphia and London: W. B. Saunders Company. 1906. (Cloth, $6.00; half morocco, $7.00, net prices).

---

A Textbook upon the Pathogenic Bacteria. For Students of Medicine and Physicians. By Joseph McFarland, M.D., Professor of Pathology and Bacteriology in the Medico-Chirurgical College, Philadelphia. New (5th) edition. Octavo volume of 647 pages, fully illustrated, a number in colors. Philadelphia and London: W. B. Saunders Company, 1906. (Cloth, $3.50 net).

---

A Textbook of Diseases of Women. By J. Clarence Webster, M.D. (Edin.) F.R.C.P.E., F.R.S.E., Professor of Obstetrics and Gynecology in Rush Medical College, in affiliation with the University of Chicago. Large octavo of 712 pages, with 372 text-illustrations and 10 colored plates. Philadelphia and London: W. B. Saunders Company. 1907. (Cloth, $7.00; half morocco, $8.00, net prices).

---

Diseases of the Lungs, by Robert H. Babcock, A.M., M.D., author of Diseases of the Heart and Arterial System. Octavo, pp. 829. With twelve colored plates and 104 text illustrations. New York and London: D. Appleton and Co. 1907. (Price $6.00).

The Elements of the Science of Nutrition. By Graham Lusk, Ph.D., M.A., F.R.S. (Edin.), Professor of Physiology at the University and Bellevue Hospital Medical College, New York. Octavo of 326 pages, illustrated. Philadelphia and London: W. B. Saunders Company, 1906. (Cloth, $2.50 net).

---

Materia Medica for Nurses. By Emily M. A. Stoney, Superintendent of the Training School for Nurses at the Carney Hospital, South Boston, Mass. 12mo of 300 pages. Third edition, thoroughly revised. Philadelphia and London: W. B. Saunders Company, 1906. (Cloth, $1.50 net).

---

The Immediate Care of the Injured. By Albert S. S. Morrow, M.D., Attending Surgeon to the Workhouse Hospital and to the New York City Home for the Aged and Infirm. Octavo of 340 pages, with 238 illustrations. Philadelphia and London: W. B. Saunders Company, 1906. (Cloth, $2.50 net).

---

Biographic Clinics. By George M. Gould, M.D., Editor of American Medicine. Vols. IV and V. Influence of Visual Function, Pathologic and Physiologic, upon the Health of Patients. 12mo, about 400 pages each. Philadelphia: P. Blakiston's Son & Co. (Price, $1.00 each, net).

---

Manual of Clinical Chemistry, by A. E. Austin, M.D., Professor of Chemistry and Toxicology in the Medical Department of Tufts College, Boston. 12mo, pp. 278. Boston: D. C. Heath & Co. 1907. (Price, $1.75).

---

Annual Report of the Surgeon-General of the Public Health and Marine Hospital Service of the United States for the fiscal year 1906. Washington: Government Printing Office.

---

A Manual of Pathology. By Guthrie McConnell, M.D., Pathologist to the St. Louis Skin and Cancer Hospital and to St. Luke's Hospital, St. Louis, Mo. 12mo of 523 pages, illustrated. Philadelphia and London: W. B. Saunders Company. 1906. (Flexible leather, $2.50 net).

---

Thornton's Pocket Medical Formulary. New (8th) edition, revised to accord with the new U. S. Pharmacopeia. Containing about 2,000 prescriptions with indications for their use. Lea Brothers & Co., Philadelphia and New York. 1907. (Leather, $1.50 net).

---

Essentials of Obstetrics. By Charles Jewett, M.D., Professor of Obstetrics and Gynecology, in the Long Island College Hospital, Brooklyn, N. Y. Third edition, thoroughly revised. 12mo, 413 pages, with 80 engravings and 5 colored plates. Philadelphia and New York: Lea Brothers & Co. 1907. (Cloth, $2.25, net).

---

Saunders's Hand-Atlases. Atlas and Epitome of Dentistry. By Prof. Gustav Preiswerk, of Basil. Edited, with additions, by Geo. W.

Warren, M.D., Professor of Operative Dentistry at the Pennsylvania College of Dental Surgery. With 44 lithographic plates in colors, 152 text-illustrations, and 350 pages of text. Philadelphia and London: W. B. Saunders Company. 1906. (Cloth, $3.50 net),

A Textbook of Pathology. By Alfred Stengel, M. D., Professor of Clinical Medicine in the University of Pennsylvania. Fifth revised edition. Octavo of 977 pages, with 399 text-illustrations, many in colors, and 7 full-page colored plates. Philadelphia and London: W. B. Saunders Company. 1906. (Cloth, $5.00; half morocco, $6.00, net prices).

A Manual of Normal Histology and Organography. By Charles Hill, Ph.D., M.D., Assistant Professor of Histology and Embryology, Northwestern University Medical School, Chicago. 12mo volume of 463 pages, with 312 illustrations. Philadelphia and London: W. B. Saunders Company, 1906. (Flexible leather, $2.00 net).

